# Cultural adaptation of a brief motivational intervention for heavy drinking among Hispanics in a medical setting

**DOI:** 10.1186/s12889-015-1984-y

**Published:** 2015-07-30

**Authors:** Craig A. Field, José Alonso Cabriales, Robert H. Woolard, Alan H. Tyroch, Raul Caetano, Yessenia Castro

**Affiliations:** Department of Psychology, The University of Texas at El Paso, 500 W. University Avenue, El Paso, TX 79968 USA; Health Sciences Center El Paso, Texas Tech University El Paso, El Paso, TX USA; Senior Research Scientist, Prevention Research Center, Pacific Institute for Research and Evaluation, Oakland, CA USA; School of Social Work, The University of Texas at Austin, Austin, TX USA

**Keywords:** Alcohol, Brief motivational intervention, Hispanics, Injury, Medical setting

## Abstract

**Background:**

Hispanics, particularly men of Mexican origin, are more likely to engage in heavy drinking and experience alcohol-related problems, but less likely to obtain treatment for alcohol problems than non-Hispanic men. Our previous research indicates that heavy-drinking Hispanics who received a brief motivational intervention (BMI) were significantly more likely than Hispanics receiving standard care to reduce subsequent alcohol use. Among Hispanics who drink heavily the BMI effectively reduced alcohol use but did not impact alcohol-related problems or treatment utilization. We hypothesized that an adapted BMI that integrates cultural values and addresses acculturative stress among Hispanics would be more effective.

**Methods/Design:**

We describe here the protocol for the design and implementation of a randomized (approximately 300 patients per condition) controlled trial evaluating the comparative effectiveness of a culturally adapted (CA) BMI in contrast to a non-adapted BMI (NA-BMI) in a community hospital setting among men of Mexican origin. Study participants will include men who were hospitalized due to an alcohol related injury or screened positive for heavy drinking. By accounting for risk and protective factors of heavy drinking among Hispanics, we hypothesize that CA-BMI will significantly decrease alcohol use and alcohol problems, and increase help-seeking and treatment utilization.

**Discussion:**

This is likely the first study to directly address alcohol related health disparities among non-treatment seeking men of Mexican origin by comparing the benefits of a CA-BMI to a NA-BMI. This study stands to not only inform interventions used in medical settings to reduce alcohol-related health disparities, but may also help reduce the public health burden of heavy alcohol use in the United States.

**Trial registration:**

Trial registration clinicaltrials.gov identifier NCT02429401; Registration date: April 28, 2015.

## Background

### Drinking among Hispanics

Research indicates that heavy drinking and alcohol problems are highest among Hispanic men compared to men of other racial/ethnic groups in the United States [1]. Compared to non-Hispanics, Hispanics have higher rates of self-reported driving while intoxicated and arrests for driving under the influence of alcohol [2]. Hispanics are more likely than non-Hispanics to report workplace, legal, social and alcohol use related health problems [3–5]. Hispanics of Mexican origin have among the highest rates of heavy drinking, driving under the influence of alcohol, arrests for driving while intoxicated, alcohol abuse, and dependence [6–8]. Among Hispanics, men are more likely to drink and drink more heavily compared to women [9, 10], and men of Mexican origin are particularly vulnerable to alcohol problems [11, 12]. Despite the increased need for treatment of alcohol problems among Hispanics in the U.S., Hispanics are less likely than non-Hispanic whites to receive specialty treatment or multiple episodes of care [9, 13–15]. Explanations for the unmet need for alcohol-related treatment in the Hispanic population include factors such as immigration experiences, racial/ethnic discrimination, insufficient availability of bilingual clinicians, low socioeconomic status, and lack of or insufficient health insurance coverage [9]. Given the prevalence of alcohol problems among men of Mexican origin, there is a strong need for evidence based interventions that are culturally and linguistically responsive.

### Brief motivational intervention among Hispanics

Brief motivational intervention (BMI) is based on motivational interviewing (MI), which is a person centered, collaborative conversation style aimed at strengthening a person’s motivation and commitment to change by addressing ambivalence about change [16]. BMI following admission for treatment of an alcohol related injury is a conversation lasting about 25 min during which the clinician adheres to MI principles and the use of core clinical tasks associated with brief interventions [17–19]. In the first study sufficiently powered to evaluate ethnic differences in response to BMI targeting heavy drinking and its associated problems, we found that BMI was more effective than standard care with heavy-drinking Hispanics (82 % Mexican or Mexican American; 89 % male) admitted for medical treatment of an alcohol-related injury and/or whom recently engaged in heavy drinking [20]. Despite more severe alcohol problems and limited treatment utilization at baseline, Hispanic participants significantly benefited from BMI in terms of alcohol use compared to non-Hispanic participants [20]. However, BMI did not influence alcohol problems or treatment utilization [20].

We hypothesized that BMI did not significantly affect alcohol problems and treatment utilization because it did not address unique stressors, social context, and cultural values that may influence alcohol-related outcomes among Hispanics [21, 22]. Subsequently, we found that Hispanic patients receiving BMI from a Hispanic provider, and patients with lower levels of acculturation were more likely to benefit from BMI compared to those who received BMI from a non-Hispanic provider or were more acculturated [21]. Patient—provider ethnic concordance may have impacted the effectiveness of the intervention, for example, by implicitly adhering to culturally appropriate modes of communication. Further, less acculturated patients may have been more responsive to such modes of communication [21, 23].

### Necessity of cultural adaptations to brief motivational intervention

The need to culturally adapt behavioral interventions has been extensively acknowledged and justified [24–26], particularly if an evidence-based intervention is insufficiently successful in changing clinical outcomes for a particular ethnic group [27]. There is widespread agreement that more effective alcohol interventions could be developed for Hispanics by taking into account cultural values (e.g., familism) and acculturation experiences (e.g., acculturative stress) specific to Hispanics [28–30]. The necessity for comparing a culturally adapted brief motivational intervention (CA-BMI) to a non-adapted intervention among heavy drinking Hispanics is consistent with the recommendation by Miller and colleagues that such research constitutes an advance in the development of optimal approaches for treating understudied groups (e.g., Mexican-Americans) [31]. Despite the need for culturally adapted alcohol interventions among Hispanics [9, 15, 32], no comparative effectiveness study has empirically evaluated a culturally adapted BMI (CA-BMI) against a non-adapted BMI (NA-BMI). As such, this paper describes the design and implementation of a CA-BMI targeting heavy-drinking men of Mexican origin. The CA-BMI will target risk and protective factors that are especially relevant to this population.

Among Hispanics, acculturative stress has been identified as an important risk factor for drinking [8, 33–37] whereas familism has been found to be an important protective factor for reducing alcohol use [38–41], and increasing help seeking and treatment utilization [42–44]. We hypothesize that a CA-BMI that directly and consistently addresses acculturative stress, familism, and other cultural factors deemed relevant through formative work with the priority population and stakeholders, as described below, will result in significant reductions in alcohol problems and increases in help-seeking and treatment utilization among Hispanics, in addition to the reductions in alcohol use already observed with the NA-BMI.

### Process for culturally adapting brief motivational intervention

#### Community advisory board

For this study we will adopt the integrative practice framework, which identifies a continuum of stakeholder engagement processes from initial solicitation of participation to maintenance [45]. The concept of *best processes* for forming, operating, and maintaining is based on Green’s recommendations [46]. Key stakeholders will include patients and their families, health care providers, behavioral health specialists, and community treatment providers. Special emphasis will be placed on engaging stakeholders with expertise in working with Hispanic persons with alcohol problems. Through the active engagement of the community advisory board (CAB) throughout the proposed project we anticipate greater influence of patients and key stakeholders on the design, implementation, and interpretation of the study. In particular, the engagement of a CAB comprised of key stakeholders is critical to the refinement of the cultural adaptation initially proposed by study investigators.

Thus far, the CAB has been instrumental in developing the individual interview guides (see below) by helping researchers prioritize the questions of interest and asking them most effectively. The CAB has also aided in the interpretation of findings from the results of the individual interviews. Further, they helped researchers develop a comprehensive community guide to treatment options which informed making referral to treatment when appropriate. This reference materials reflect a broad range of services which may be of benefit to Hispanic males who were recently injured and engage in at risk drinking. In addition to medical detoxification, inpatient and outpatient services and alcoholics anonymous, these referrals included general counseling, physical and occupational rehabilitation, employment commission and other social services. The CAB has also provided feedback on the intervention itself including ways in which to be more culturally sensitive and responsive in addition to the adaptation itself. This was achieved, in part, by the development of videos reflecting the NA-BMI and potential components of the CA-BMI. In addition, the CAB has provided feedback on the selection of patient materials including psychoeducational information regarding heavy drinking and behavior change.

As part of study preparation and intervention adaptation, we will employ procedures consistent with recent models for intervention adaptation [29, 47], as was recently done by Castro and colleagues to adapt an evidence-based and theoretically driven motivational intervention for multiple cancer-risk behaviors among Hispanic smokers [48]. They used expert consultation, focus groups with the priority population, pretesting of program materials, and pilot testing of the intervention to culturally adapt an intervention being evaluated in a randomized controlled trial [48, 49].

#### Individual interviews and pretesting of materials

To further inform the CA-BMI, individual interviews will be conducted with at least 25 Mexican or Mexican American men recently admitted for an injury where they were drinking prior to the injury or engage in heavy drinking. Interviews will be facilitated in English and Spanish and sessions will last approximately 1 ½ hours. Trained interviewers will use a structured open-ended interview guide developed by the investigators, other study staff trained in qualitative methods, and CAB members. Questions are intended to inform adaptations by exploring issues around alcohol use, and treatment-seeking that are important to the priority population, and may need to be addressed in the CA-BMI. All interviews will be recorded and transcribed; transcripts will be coded by two individuals to capture salient themes using content analysis [50]. Interviews will also be used for pretesting of all potential study materials. Participants will provide feedback on the acceptability, appeal, preference, and usefulness of materials.

#### Pilot testing

After incorporating changes as a result of the individual interviews, CAB feedback and stakeholder feedback, the intervention protocol will be tested with 10 participants. Based on the pilot testing, counselors and patients will provide additional feedback on the intervention protocol. During piloting testing, the BMI and instruments will be tailored to better fit patients’ responses during pilot assessment, and BMI sessions.

### Accounting for patient and provider perspectives

The proposed study will measure a number of patient-centered outcomes. Alcohol use and alcohol problems have a significant impact on patients, their family and social networks, the community, and healthcare system. While these outcomes are of interest to patients and evaluators alike, treatment utilization and help-seeking are particularly important patient outcomes of interest. Based on feedback from patients, healthcare providers, behavioral health specialists, and community providers, we are broadening our definition of treatment utilization to include help-seeking from informal or cultural agents of behavior change that are likely to be accessed by Hispanics (e.g., church leaders, folk healers) [51].

The proposed study will also assess the feasibility and acceptability of the intervention from the perspectives of the patients and healthcare providers. Both NA-BMI and CA-BMI are based on MI principles and, therefore, explore patient preferences, and perspectives to develop a plan for change. As a result, we recognize the influence that the patient’s perspective of the intervention may have on targeted outcomes and will incorporate a measurement of patient satisfaction. We will be able to explore whether high ratings of satisfaction predict better outcomes from BMI. We will also be able to explore whether CA-BMI leads to greater patient satisfaction. The influence of patient satisfaction with BMI sessions on patient outcomes or a comparison across intervention strategies has not been routinely reported, as we propose here.

Ultimately, the dissemination and implementation of culturally adapted brief interventions in trauma care and emergency department settings rely upon organizational factors-most importantly, the perspective of healthcare providers. As a result, we will also assess the perceptions of CA-BMI and NA-BMI among healthcare providers as key stakeholders. First, the acceptability and feasibility of both interventions will be assessed prior and after patient recruitment. Evaluations of provider perspectives on the applicability, relevance, and potential adoption of a CA-BMI will be conducted. In this way, we can explore the impact of implementation of the protocol on provider perspectives and take into account the potential barriers and facilitators of dissemination and implementation. Second, like patient satisfaction, provider ratings of BMI will be assessed following CA-BMI and NA-BMI.

## Methods/Design

### Participants

#### Study site and recruitment

At present, approval has been obtained from the Institutional Review Boards from both The University of Texas at El Paso and University Medical Center (UMC) of El Paso. All data collection will be conducted at UMC of El Paso in El Paso, Texas. The ethical guidelines for research with human subjects followed by the present study protocol are compliant with the Helsinki Declaration. UMC is the largest public hospital (not-for-profit) located on the U.S./México border. It is a 396-bed, licensed, acute care facility and the only teaching hospital in the West Texas/Southern New Mexico region. UMC is also the only Level I Trauma Center within a 280 mile radius of El Paso. Eighty five percent of all trauma cases in the region annually are transported to UMC. In 2014, approximately 2,750 trauma patients were admitted to the UMC Trauma Center and approximately 75 % were Hispanic. The Emergency Department at UMC is also one of the busiest in the region, treating more than 55,000 patients annually, of whom approximately 80 % are Hispanic.

### Design and procedure

**Fig. 1:**
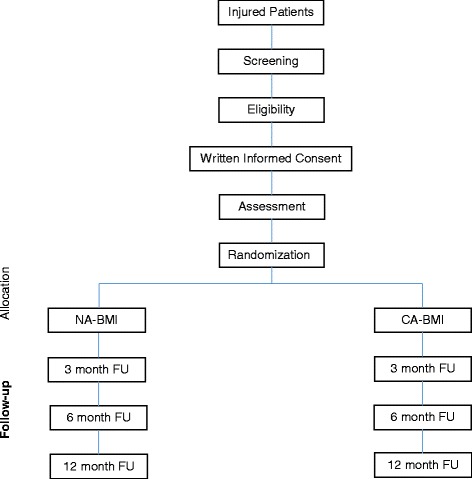
CONSORT diagram of participant flow

In this comparative effectiveness study, we will recruit 600 English- or Spanish-speaking heavy-drinking men of Mexican origin who are admitted to the hospital for medical treatment of an alcohol-related injury or heavy drinking. Participants will be randomized to receive a CA-BMI or NA-BMI. The primary outcomes of interest are alcohol use, alcohol problems, help-seeking, and treatment utilization. Follow-up assessments will be completed at 3, 6, and 12 months post-treatment. See Fig. 1.

#### Screening

The UMC Clinical Coordinator and Screening Coordinator, in collaboration with other study staff, will ensure that comprehensive and standardized screening procedures are used to identify potentially eligible patients. At UMC, patients screen positive for an alcohol-related injury or heavy drinking based on 1) positive blood alcohol concentration (BAC), 2) initiation of a medical protocol for alcohol detoxification, or 3) a score ≥ 4 on the first three items of the Alcohol Use Disorders Identification Test (AUDIT-C), which is a gender-specific cut-off for heavy drinking among men. Based on current medical standards, all injured patients who have blood drawn have their BAC tested as part of routine laboratory tests. Once medically stable, all injured patients are screened by the UMC Screening Coordinator using the AUDIT-C, a standardized, internationally validated self-report measure of alcohol use, and heavy drinking [52, 53].

#### Inclusion/exclusion criteria

Eligibility criteria for this study are the following: 1) aged 18 or older, 2) treated for injury associated with a motor vehicle collision (involving driver, passenger, or pedestrian), a violence-related injury (i.e., gunshot, stab wounds, other assault related injuries), or a fall based on e-codes from the 10^th^ edition of the International Classification of Diseases, and 3) screen positive for an alcohol-related injury or heavy drinking as described above. Exclusion criteria are: 1) traumatic brain injury as indicated by a Glasgow Coma Scale score < 15 [54], or 2) cognitive impairment as indicated by a score of ≤ 24 on the Mini-Mental Status Exam (MMSE) [55, 56]. Patients who do not successfully complete the MMSE will be monitored regularly by the Screening Coordinator to reassess participation. Intoxicated patients at admission will be approached during their hospital stay once medically stable.

#### Ethnic identification

Ethnicity will be confirmed by study staff using procedures similar to general population surveys (i.e., 2010 census). Response options include: 1) no, not of Hispanic, Latino, or Spanish origin, 2) Yes, Mexican, Mexican American, Chicano, 3) Yes, Puerto Rican, 4) Yes, Cuban, or 5) Yes, another Hispanic, Latino or Spanish origin [57]. Patients who identify themselves of Hispanic, Latino, or Spanish origins, and only describe themselves as Mexican, Mexican American, or Chicano will be eligible to participate. In our prior study, 82 % of Hispanics identified themselves as Mexican, Mexican American, or Chicano only [20].

#### Randomization procedures

After obtaining informed consent, patients will complete the baseline assessment (described below). Subsequently, participants will be randomized to the CA-BMI or NA-BMI. Study staff will be blind to treatment assignment during the baseline assessment, and staff responsible for the assessment will not provide the intervention (vice versa). We will use an adaptive randomization procedure that minimizes imbalances in covariates [58] using a SAS macro [59]. This procedure ensures adequate distribution of key characteristics (i.e., acculturation, nativity, language of assessment) which have been found to influence outcomes [21].

### Brief motivational interventions

Both interventions (NA-BMI and CA-BMI) will adhere to MI principles [16, 17, 60], and practice of BMI. To help maintain the integrity of the two interventions, clinicians will be trained and supervised in the provision of only one of the interventions (NA-BMI or CA-BMI). As part of standard BMI protocols, all participants will receive personalized feedback based on results from their screening, as well as a personal drinking profile based on the results of the baseline assessment [61, 62].

#### Non-adapted brief motivational intervention (NA-BMI)

Consistent with MI, the core NA-BMI components include: 1) personalized feedback based on screening and baseline assessment, 2) exploring decisional balance of alcohol use from patient’s perspective, 3) building motivation for change through the assessment, and discussion of patients’ self-report of levels of importance, confidence, and readiness to change, 4) enhancing commitment to change by exploring patient’s options for change, and developing a change plan if indicated, and 5) providing alcohol treatment referrals. The NA-BMI will not target cultural risk or protective factors beyond any normal tailoring that may occur in standard BMI. Personalized feedback will be based on U.S. general population drinking norms, and frequency of alcohol problems.

#### Culturally adapted brief motivational intervention (CA-BMI)

CA-BMI also adheres to the core principles of both MI and BMI. In CA-BMI, core components of BMI are adapted to be responsive to the unique risk (i.e., acculturative stress) and protective (i.e., familism) factors associated with heavy drinking, alcohol problems, help-seeking, and treatment utilization in Hispanics. CA-BMI goes beyond any tailoring that may occur in NA-BMI by targeting important predictive factors of drinking in Hispanics. Two *primary* adaptations to CA-BMI will be made.

First, CA-BMI will incorporate the assessment, and personalized feedback on the impact of acculturative stress on drinking in order to decrease temptation to drink, and increase confidence to avoid drinking. Participants will receive feedback about the types, and intensity of acculturative stress they may experience (e.g., immigration-related issues, cultural congruity), and clinicians will evoke the relation of acculturative stress to temptation, and confidence to avoid drinking. Second, CA-BMI will integrate family, and community as reasons for change, and as agents of behavior change when considering the impact of drinking, plans for changing drinking, and engagement in help-seeking behaviors. Similar to methods developed by Lee et al. (2011) [63] and Añez et al. (2008) [64], the CA-BMI will incorporate a discussion of how social context and family dynamics may affect drinking. Feedback, and a discussion of social pressures (e.g., cultural gatherings) to engage in heavy drinking as well as changes in drinking patterns (e.g., concerns about drinking from family) will also be included. When developing a change plan, there will be an explicit discussion, and elaboration of changes in family or community networks that may facilitate reductions in drinking. Participants will be encouraged to identify, and actively engage family and community members as helpers in their efforts to change their drinking. For example, they may identify a respected member of the family or community with whom they can talk about their commitment to change. Participants’ cultural norms, expectations, and personal values will be discussed to illuminate discrepancies with current drinking behavior, which may increase motivation to change (see Añez et al., 2008 [64]).

These two central modifications result in a culturally adapted intervention that is substantially distinct in content, and focus from a non-adapted intervention, while still maintaining consistency with MI, and its application within brief alcohol interventions. Based on adaptations, we anticipate the potential mediators or mechanisms of behavior change specific to CA-BMI to be: 1) temptation to drink and confidence to avoid drinking, and 2) increased support from family, and friends in general as well as specific support to change drinking, and seek treatment. We will evaluate a definition of treatment utilization that is more comprehensive than that in our prior study, which assessed the use of formal inpatient/outpatient substance abuse treatment, and attendance to self-help groups such as Alcoholics Anonymous [20]. We will assess engagement in formal treatment networks as well as informal help-seeking common among Hispanics (e.g., family, religious leaders).

### Training and treatment fidelity

#### Interventionists

We have determined that Hispanics respond more positively to BMI, and that patient—provider ethnic matching significantly influences drinking outcome above and beyond the effect of the intervention. Both NA-BMI and CA-BMI will be provided by bilingual, bicultural, and same gender interventionists. Therefore, gender, ethnicity, and ability to speak both Spanish, and English will be held constant across interventions. By holding these three factors constant we can determine the differential influence of the two interventions on alcohol-related outcomes. To avoid contamination, providers of CA-BMI and NA BMI will be trained, and supervised separately throughout the study period. Study staff who are responsible for the intervention will not be responsible for baseline or follow-up assessments.

#### Interventionist training and supervision

Dr. Field, the Principal Investigator (PI), will be primarily responsible for oversight of the training, and supervision of interventionists. Dr. Field has received advanced training in MI (including the use of treatment fidelity measures for supervision), and is a member of the Motivational Interviewing Network of Trainers (MINT). Dr. Castro, a Co-investigator (Co-I), will facilitate training, and supervision. Dr. Castro is a bilingual, bicultural clinical psychologist with expertise in culturally sensitive service provision, and extensive experience in providing Spanish-language services to Hispanic populations. Dr. Castro has experience with cultural adaptations of smoking cessation interventions based on MI [49]. Training manuals for CA-BMI will be adapted from existing brief intervention manuals from previous trials (R01-AA13824; R01-AA015439) conducted by the PI. As part of the training process, the PI’s research team has developed a set of training videos that will serve for the purposes of both NA-BMI and CA-BMI training. These short videos portray typical BMI sessions through role plays including study investigators providing BMI. Prior to training, interventionists will read *Motivational Interviewing* [16], read the intervention manual (CA-BMI or NA-BMI), and watch the aforementioned videos. A two-day interactive training on MI principles, and the practice of brief interventions will be provided by a bilingual trainer who is a member of the MINT. In addition to the study PI and Co-I, a Training Coordinator, Patricia Juárez will be primarily responsible for the training and supervision of interventionists. Ms. Juárez has received advanced MI training, and is also a member of the MINT. Supervision will be provided by Ms. Juárez throughout the study, and will include the following: 1) weekly supervision, 2) on-site observation, feedback, and/or coaching sessions (at least one per month), and 3) a one-day booster training provided every six months. This provides trainees the opportunity to practice, receive feedback, and refine their skills.

#### Treatment fidelity

Procedures to ensure treatment fidelity are based on recommendations from the NIH Behavior Change Consortium [65], which established best practices in treatment fidelity of behavioral interventions. Fidelity will consist of two components: 1) *adherence*, or whether the interventionist carried out specified procedures of CA-BMI or NA-BMI, and 2) *competence*, or the interventionist’s level of skill in implementing the intervention using MI. Adherence to CA-BMI or NA-BMI protocols, and competence in MI will be assessed throughout the study by reviewing and coding recorded interventions.

#### Adherence to CA-BMI and NA-BMI

Intervention protocols will be codified using behavioral checklists, which have been found to be useful in monitoring intervention delivery [66]. These will be based on checklists from our prior studies (R01- AA13824; R01-AA015439 and R01-DA026088). The CA-BMI checklist will be adapted from a current checklist used to assess BMI adherence [19]. Interventions will be coded using a checklist based on prescribed, and proscribed components of the respective BMI. Adherence checklists will be used in supervision, and to establish adherence and discriminability between CA-BMI, and NA-BMI.

#### Competence in MI

Interventionists’ competence will be assessed in both BMIs using the Motivational Interviewing Treatment Integrity (MITI) v3.1 [67]. The MITI is a behavioral coding system that measures competence in MI by assessing the degree to which therapist behaviors are adherent or non-adherent to MI. Supervisors will complete the MITI on audio recordings of BMIs throughout the recruitment period. The MITI will be used to derive summary scores with benchmarks for basic, and expert proficiency in MI. We will use expert-level performance as the threshold for establishing, and maintaining competency. MITI summary scores will be used to provide feedback during supervision, and to demonstrate competence in MI.

#### Assessment of patient-oriented outcomes

This study will employ bilingual staff to conduct the assessments, which facilitates the option of reading questions aloud to participants, thereby assisting patients to complete the in-person baseline assessment during their hospital stay [68]. All measures used were developed or tested in Spanish, and are available in both Spanish, and English. Staff responsible for baseline and follow up assessments are blind to participants’ intervention condition, and will be trained and supervised throughout the recruitment period.

### Measures

#### Drinking outcomes

*The Timeline Follow Back (TLFB)* will be used to obtain data on the prior month’s days of alcohol use, calculated average weekly drinks, days of binge use, average number of drinks per drinking day, maximum number of drinks on any day, and drinking above at-risk levels [69, 70]. *The Short Inventory of Problems (SIP + 6)* will be used to assess the number of alcohol-related consequences as an outcome variable. The SIP + 6 is a brief version of the Drinking Inventory of Consequences (DrInC) [71]. The SIP + 6 contains five 3-item scales related to consequences in the physical, social responsibility, intrapersonal, impulse control, and interpersonal domains. This scale has been validated with both English and Spanish speaking Hispanics [72].

#### Treatment utilization

*The Treatment Services Review (TSR)* will be used to collect information regarding the receipt of services for seven potential problem areas—medical status, employment and support, drug use, alcohol use, legal status, family/social status, and psychiatric status [73, 74]. To complement this measure, the *Mexican American Prevalence and Services Survey (MAPS)* will be used to assess help seeking [51] through other methods, such as chiropractor, homeopath, minister, folk healer (i.e., curandero), spiritualist, santero, and sobador.

#### Social support

Social support will be measured using four subscales including social cohesion, support from friends and family, and family cultural conflict [75]. The *Important People and Activities (IPA)* interview will complement the assessment of general social support by assessing social support specific to alcohol use [76, 77]. The IPA assesses support from family and friends through both structural, and functional processes. The IPA also assesses how people within the patient’s social network would react to the patient’s drinking on a five point Likert type scale ranging from 1 (*left or made you leave*) to 5 (*encouraged your drinking*).

#### Temptation and confidence

Situational temptation to drink will be measured using the 20 item *Temptation to Drink Scale*. This measure assesses how tempted an individual is to engage in a variety of health behaviors such as substance use. Confidence to avoid drinking will be measured using the 20 item *Abstinence Self-efficacy Scale*. This scale assesses an individual's confidence to abstain from drinking alcohol in various situations. Both scales assess temptation to drink or confidence to avoid drinking within these contexts: negative affect, social/positive, physical, and other concerns, and cravings/urges which may precipitate drinking [78, 79].

#### Readiness to change

*The Readiness to Change Questionnaire (Treatment Version; RCQ [TV])* assesses a patient’s level of readiness to change their alcohol use (e.g., reduce, quit) with respect to the precontemplation, contemplation, and action stages of change [80]. The RCQ (TV) includes 12 Likert-type items (3 per stage) ranging from −2 (*Strongly disagree*) to +2 (*Strongly agree*). A score is computed per subscale/stage, and the participant is assigned to the one with the highest score (in case where two or more scores are the same, participants are assigned to the stage that is furthest along the staging continuum [i.e., closer to action]).

#### Acculturation

Acculturation will be measured with the *Multidimensional Acculturation Scale II* (MAS II) [81]. The MAS II includes 22 items (from 0 [*does not apply to me*] to 5 [*very much/very well*]) assessing 4 factors: English proficiency, Spanish proficiency, identification with Mexican cultural identity, and identification with American cultural identity. MAS II factors have been found reliable (α = .78-.93).

#### Acculturative stress

Acculturative stress will be measured with the *Multidimensional Acculturative Stress Inventory (MASI),* which lists situations that Hispanics may have experienced within past 3 months [82]. The MASI includes 36 items ranging from 1 (*never*) to 5 (*daily or almost daily*). Identified factors are: Spanish competency pressures, English competency pressures, pressure to acculturate, and pressure against acculturation. Subscales (αs = .77 to .93), and the overall scale have demonstrated adequate reliability levels (α = .90) [82].

#### Therapeutic alliance

*The Helping Alliance Questionnaire (HAQ-II)* is part of the Penn Helping Alliance Scales [83, 84]. This self-report scale assesses experiences in therapy from both the patient and therapist's perspective. Thus, there is a patient version, and a therapist version. The HAQ-II includes 11 Likert-type items from 1 (*completely disagree*) to 4 (*completely agree*).

### Approach to analyses

Analyses investigating group differences in alcohol problems, and treatment utilization will use random coefficient models [85, 86]. Longitudinal models will use the following sequence of steps recommended by Singer and Willett [86]: 1) examine empirical growth plots, 2) fit an unconditional means model, 3) fit an unconditional linear growth model, 4) fit an unconditional non-linear model, 5) determine the best model of longitudinal change by comparing models in the previous two steps using the Akaike information criterion (AIC), 6) select the most appropriate error covariance structure using AIC, and 7) add level-2 predictors (e.g., intervention group).

Potential moderators will be examined by constructing interaction terms between treatment, and a priori moderator variables (e.g., acculturative stress and familism) to examine the possibility that the relation between a putative moderator, and outcome differ across treatments [87]. Mediation analysis (per recommendations by MacKinnon [2008]) will be conducted using a growth-curve framework implemented in an SEM [88]. Models will be constructed by first fitting growth models for mediators, and outcomes, and then fitting mediational growth models. Latent growth models will be comprised of at least two latent factors; one factor will represent the initial status, and one or more factors will represent the growth rate of a variable. The growth factor of the mediator will be regressed on the initial status of the mediator, the outcome, and the intervention group. A significant intervention effect establishes a relation between the intervention group, and the mediator, controlling for baseline levels of the mediator and outcome. Next, the growth factor will be regressed on the initial status of the mediator, the outcome, the slope of the mediator, and the intervention group. A significant effect of the mediator growth factor establishes a relation between change in the mediator, and change in the outcome, controlling for baseline levels of mediators and outcome.

### Power analysis

Power for the hypothesized intervention effects was estimated using Monte Carlo studies. For longitudinal mixed models, a simulation was conducted (per example by Gelman and Hill) [89] using the *R* software, and power for mediation models was estimated using simulations in the *Mplus* software [90]. Culturally adapted interventions for other mental health problems have produced effect sizes of .45 [91]. The proposed effect size is informed by our previous study, which produced effect sizes between .25 and .32 with an average effect size of .28 [20, 92]. For this study, we estimated effect sizes equivalent to *d* = .28 for continuous outcomes and *h* = .28 for binary outcomes (nearly a 50 % increase in treatment utilization), using effect sizes for mixed models that are equivalent to these classical effect sizes [93].

Power estimates were derived as the proportion of significant effects (two-tailed α = .05) across like simulations [94]. We simulated 10,000 data sets of 400 observations under the assumption that missing data will be ameliorated with the strategies described in the missing data section, and all participants will contribute data to the analysis. Intraclass correlation was simulated at .65 based on the intraclass correlations observed in our prior study. Power was .81 for continuous outcomes, and .83 for binary outcomes. A power analysis for a moderation effect by simulating a three-way interaction between treatment, time, and a moderator (e.g., familism) was conducted. Per Kraemer and colleagues’ recommendations, the interaction was examined using plots, confidence intervals, and *p* values [95]. We investigated the power for a hypothesized contrast at high, and low levels of a moderator (e.g., low v. high acculturative stress in participants in CA-BMI) through a simple slope contrast at 1 SD ± the moderator mean for participants [96]. Power for an effect size equivalent to *d* = .29 was .78 for a continuous outcome, and power for an effect size equivalent to *h* = .27 was .82 for a binary outcome. Power analysis for the proposed mediation model was conducted using a Monte Carlo simulation [90]. Using standardized regression coefficients, we conducted a simulation for 10,000 data sets in which the path from the intervention to the mediator was .12 (equivalent to *d* = .22), and the path from the mediator to the outcome was .30 for standardized variables, reflecting the stronger relation that we anticipate between the mediators and outcomes. The Monte Carlo simulation indicated that power for the indirect effect was .77. Thus, accounting for a potential 20 % participant attrition, the proposed sample size should provide sufficient power for all planned analyses.

## Discussion

Given the increased prevalence and disproportionate impact of heavy drinking and alcohol problems among Hispanic men in the United States, particularly Mexican-origin men, and the corresponding unmet need for alcohol-related treatment, it is essential that we address alcohol-related health disparities in this at-risk, underserved and growing population [97]. Particularly, studies suggest that racial/ethnic disparities in treatment utilization remain evident even after controlling for differences in insurance coverage, income, and education [14, 98]. Our previous work demonstrated that Hispanic patients benefit more from BMI with respect to decreased alcohol use but not alcohol related problems or treatment utilization [20, 21]. Additionally, patients who received the BMI from a Hispanic provider, and those who demonstrated lower levels of acculturation, were significantly more likely to benefit from the intervention than those who received the intervention from a non-Hispanic provider or demonstrated higher acculturation, respectively, strongly suggesting the role of cultural factors [21]. Moreover, a meta-analysis indicated that mental health interventions that account for cultural context and values are four times more effective than non-adapted interventions [91]. Taken together, these findings point toward the promise of a CA-BMI that incorporates cultural factors like familism, and acculturative stress, as in the present study. Yet, no known published study to date has compared the effectiveness of an alcohol CA-BMI in relation to a NA-BMI among non-treatment seeking, heavy drinking Mexican-origin men.

The potential for dissemination and implementation of a CA-BMI in the trauma care setting is high. The proposed comparative effectiveness trial is consistent with the call by the American College of Surgeons to provide screening and brief intervention to heavy-drinking injured patients [99]. Since 2006, the American College of Surgeons and Committee on Trauma has required that all Level I Trauma Centers have the capacity to identify injured patients with alcohol problems and provide BMI. From a behavioral health perspective, BMIs are critical components as part of the services provided in trauma care settings. Within medical settings, BMIs seem to be efficacious in part due to the window of opportunity for identifying non-treatment seeking heavy drinkers and a teachable moment for those involved in an alcohol-related injury.

A culturally adapted intervention may be more effective and better received as an alternative to currently available interventions. Patient satisfaction and provider preference will likely have significant impact on the practice of BMIs in these settings even if a CA-BMI is not superior to a NA-BMI in terms of drinking outcomes. This would be particularly true if patients and providers prefer a CA-BMI to a NA-BMI despite their equivalent efficacy or similar costs. The findings of this study will be particularly pertinent to trauma centers in states like California, Arizona, New Mexico and Texas which provide care to a largely Hispanic patient population. Moreover, given the advent of telehealth, it may also have important implications for other locations where there is a small underserved, Hispanic population. These facilities may not have the human or financial resources to provide intervention to a small yet underserved population of Hispanics. Through telehealth, CA-BMI can be provided in locales with emerging bilingual, bicultural Hispanic populations thereby increasing the public health impact of CA-BMI on the reduction of alcohol related health disparities.

Although the potential for dissemination and implementation of the findings from the proposed study are high based on the previously noted factors, it is not without potential barriers. According the World Health Organization (WHO), barriers to conducting SBI include a lack of knowledge and skills, lack of time, lack of financial benefits, lack of professional benefits, lack of diagnostic support, and the overarching organization of the health care system [100]. The proposed study will consider strategies that have been outlined previously [101–103] in order to overcome barriers and effectively implement the proposed BMI within the trauma department. Based on prior experience, our team recognizes that even highly invested trauma centers must overcome fiscal and human resource barriers to meeting the unfunded mandate of a Level I Trauma Center set by the Committee on Trauma [104]. However, we remain confident that through the engagement of key stakeholders including patients, providers and community these barriers can be overcome and CA-BMI will become an evidence-based best practice in trauma centers and emergency departments.

## Conclusion

There exists an unmet need for the treatment of heavy alcohol use, and alcohol-related problems among men of Mexican origin. This paper proposes the first comparative effectiveness study regarding the cultural adaptation of a BMI compared to a NA-BMI in the medical setting among heavy drinking men of Mexican origin. The successful adaptation and implementation of this CA-BMI may contribute to reducing the public health burden of heavy alcohol use in the United States.
